# Supplementary material for: Unveiling the Bioactive Potential of the Invasive Jellyfish Phyllorhiza punctata Through Integrative Transcriptomic and Proteomic Analyses
Source: Biomolecules. 2025 Aug 4;15(8):1121. doi: 10.3390/biom15081121 (PMC12383608; doi:10.3390/biom15081121)
Supplement: Supplementary file 1 [file biomolecules-15-01121-s001.zip › Suplementary Material/FileS3/PEPFOLD4/XP_065059114_14545/clusters.html]

 PEP-FOLD clusters


Download archive of all models  here   

|  |  |  |
| --- | --- | --- |
| # | file | sOPEP |
| # cluster 1 - Size: 1 |  |  |
|  | XP\_065059114\_14545-model1 | -4.50238 |
| # cluster 2 - Size: 62 |  |  |
|  | XP\_065059114\_14545-model2 | -4.4207 |
|  | XP\_065059114\_14545-model2.1 | -4.36995 |
|  | XP\_065059114\_14545-model2.2 | -4.30102 |
|  | XP\_065059114\_14545-model2.3 | -4.25755 |
|  | XP\_065059114\_14545-model2.4 | -4.25755 |
|  | XP\_065059114\_14545-model2.5 | -4.219 |
|  | XP\_065059114\_14545-model2.6 | -4.16274 |
|  | XP\_065059114\_14545-model2.7 | -4.1596 |
|  | XP\_065059114\_14545-model2.8 | -4.1596 |
|  | XP\_065059114\_14545-model2.9 | -4.1596 |
|  | XP\_065059114\_14545-model2.10 | -4.1596 |
|  | XP\_065059114\_14545-model2.11 | -4.14994 |
|  | XP\_065059114\_14545-model2.12 | -4.14155 |
|  | XP\_065059114\_14545-model2.13 | -4.14155 |
|  | XP\_065059114\_14545-model2.14 | -4.14155 |
|  | XP\_065059114\_14545-model2.15 | -4.13634 |
|  | XP\_065059114\_14545-model2.16 | -4.13634 |
|  | XP\_065059114\_14545-model2.17 | -4.13634 |
|  | XP\_065059114\_14545-model2.18 | -4.13549 |
|  | XP\_065059114\_14545-model2.19 | -4.11879 |
|  | XP\_065059114\_14545-model2.20 | -4.11672 |
|  | XP\_065059114\_14545-model2.21 | -4.11672 |
|  | XP\_065059114\_14545-model2.22 | -4.11672 |
|  | XP\_065059114\_14545-model2.23 | -4.09072 |
|  | XP\_065059114\_14545-model2.24 | -4.08688 |
|  | XP\_065059114\_14545-model2.25 | -4.07203 |
|  | XP\_065059114\_14545-model2.26 | -4.05845 |
|  | XP\_065059114\_14545-model2.27 | -4.03809 |
|  | XP\_065059114\_14545-model2.28 | -4.03786 |
|  | XP\_065059114\_14545-model2.29 | -4.03372 |
|  | XP\_065059114\_14545-model2.30 | -4.01753 |
|  | XP\_065059114\_14545-model2.31 | -4.01678 |
|  | XP\_065059114\_14545-model2.32 | -4.01678 |
|  | XP\_065059114\_14545-model2.33 | -4.01519 |
|  | XP\_065059114\_14545-model2.34 | -4.01058 |
|  | XP\_065059114\_14545-model2.35 | -4.00338 |
|  | XP\_065059114\_14545-model2.36 | -3.99986 |
|  | XP\_065059114\_14545-model2.37 | -3.98788 |
|  | XP\_065059114\_14545-model2.38 | -3.98068 |
|  | XP\_065059114\_14545-model2.39 | -3.97955 |
|  | XP\_065059114\_14545-model2.40 | -3.97853 |
|  | XP\_065059114\_14545-model2.41 | -3.95867 |
|  | XP\_065059114\_14545-model2.42 | -3.95831 |
|  | XP\_065059114\_14545-model2.43 | -3.94151 |
|  | XP\_065059114\_14545-model2.44 | -3.93654 |
|  | XP\_065059114\_14545-model2.45 | -3.93298 |
|  | XP\_065059114\_14545-model2.46 | -3.92625 |
|  | XP\_065059114\_14545-model2.47 | -3.89932 |
|  | XP\_065059114\_14545-model2.48 | -3.8898 |
|  | XP\_065059114\_14545-model2.49 | -3.86875 |
|  | XP\_065059114\_14545-model2.50 | -3.84746 |
|  | XP\_065059114\_14545-model2.51 | -3.82804 |
|  | XP\_065059114\_14545-model2.52 | -3.82155 |
|  | XP\_065059114\_14545-model2.53 | -3.81778 |
|  | XP\_065059114\_14545-model2.54 | -3.77066 |
|  | XP\_065059114\_14545-model2.55 | -3.73611 |
|  | XP\_065059114\_14545-model2.56 | -3.70301 |
|  | XP\_065059114\_14545-model2.57 | -3.69689 |
|  | XP\_065059114\_14545-model2.58 | -3.63644 |
|  | XP\_065059114\_14545-model2.59 | -3.5894 |
|  | XP\_065059114\_14545-model2.60 | -3.58274 |
|  | XP\_065059114\_14545-model2.61 | -3.54975 |
| # cluster 3 - Size: 29 |  |  |
|  | XP\_065059114\_14545-model3 | -4.29215 |
|  | XP\_065059114\_14545-model3.1 | -4.29215 |
|  | XP\_065059114\_14545-model3.2 | -4.28948 |
|  | XP\_065059114\_14545-model3.3 | -4.25101 |
|  | XP\_065059114\_14545-model3.4 | -4.25101 |
|  | XP\_065059114\_14545-model3.5 | -4.25101 |
|  | XP\_065059114\_14545-model3.6 | -4.25101 |
|  | XP\_065059114\_14545-model3.7 | -4.23198 |
|  | XP\_065059114\_14545-model3.8 | -4.18993 |
|  | XP\_065059114\_14545-model3.9 | -4.1894 |
|  | XP\_065059114\_14545-model3.10 | -4.1894 |
|  | XP\_065059114\_14545-model3.11 | -4.1894 |
|  | XP\_065059114\_14545-model3.12 | -4.18215 |
|  | XP\_065059114\_14545-model3.13 | -4.18215 |
|  | XP\_065059114\_14545-model3.14 | -4.17465 |
|  | XP\_065059114\_14545-model3.15 | -4.16291 |
|  | XP\_065059114\_14545-model3.16 | -4.16291 |
|  | XP\_065059114\_14545-model3.17 | -4.10786 |
|  | XP\_065059114\_14545-model3.18 | -4.08984 |
|  | XP\_065059114\_14545-model3.19 | -4.0643 |
|  | XP\_065059114\_14545-model3.20 | -4.00357 |
|  | XP\_065059114\_14545-model3.21 | -3.99906 |
|  | XP\_065059114\_14545-model3.22 | -3.99679 |
|  | XP\_065059114\_14545-model3.23 | -3.96089 |
|  | XP\_065059114\_14545-model3.24 | -3.92032 |
|  | XP\_065059114\_14545-model3.25 | -3.78784 |
|  | XP\_065059114\_14545-model3.26 | -3.71289 |
|  | XP\_065059114\_14545-model3.27 | -3.67959 |
|  | XP\_065059114\_14545-model3.28 | -3.56634 |
| # cluster 4 - Size: 1 |  |  |
|  | XP\_065059114\_14545-model4 | -3.71864 |
| # cluster 5 - Size: 1 |  |  |
|  | XP\_065059114\_14545-model5 | -3.64571 |
| # cluster 6 - Size: 2 |  |  |
|  | XP\_065059114\_14545-model6 | -3.59994 |
|  | XP\_065059114\_14545-model6.1 | -3.57773 |
| # cluster 7 - Size: 2 |  |  |
|  | XP\_065059114\_14545-model7 | -3.59709 |
|  | XP\_065059114\_14545-model7.1 | -3.55222 |
| # cluster 8 - Size: 2 |  |  |
|  | XP\_065059114\_14545-model8 | -3.59059 |
|  | XP\_065059114\_14545-model8.1 | -3.49993 |
|  |  |  |
